# Comparative Effects of Dietary Protein, Creatine, and Omega-3 Supplementation on Muscle Strength, Endurance, and Recovery in Trained Athletes: A Systematic Review and Network Meta-Analysis

**DOI:** 10.3390/nu18060909

**Published:** 2026-03-13

**Authors:** Ziyu Wang, Gang Qin, Byung-Min Kim

**Affiliations:** 1 College of Sports, Sejong University, Gwangjin-gu, Seoul 05006, Republic of Korea; 17806171816@163.com; 2 School of Physical Education, Hanyang University, Seoul 04763, Republic of Korea; qgwzy521@163.com

**Keywords:** network meta-analysis, dietary supplements, athletic performance, creatine, omega-3 fatty acids

## Abstract

This systematic review and network meta-analysis aimed to compare the effects of dietary protein, creatine, and omega-3 fatty acid supplementation on muscle strength, endurance performance, and recovery outcomes in trained athletes. A comprehensive literature search across MEDLINE, Embase, Cochrane CENTRAL, Web of Science, SPORTDiscus, and Scopus identified randomized controlled trials evaluating these supplements in individuals engaged in structured training for a minimum of six months. Network meta-analysis employing a frequentist random-effects model synthesized direct and indirect evidence, with treatment rankings determined using Surface Under the Cumulative Ranking curve probabilities. The analysis incorporated 35 trials enrolling 1211 participants. Creatine supplementation demonstrated superior effects for muscle strength (SMD = 0.46, 95% CI: 0.29 to 0.63, SUCRA = 82.4%), protein supplementation proved most effective for endurance performance (SMD = 0.28, 95% CI: 0.08 to 0.48, SUCRA = 85.2%), and omega-3 supplementation yielded the greatest benefits for recovery outcomes (SMD = 0.40, 95% CI: 0.18 to 0.62, SUCRA = 88.7%). Network consistency assessment revealed no significant disagreement between direct and indirect evidence across all outcomes. These findings reveal an outcome-specific efficacy pattern supporting targeted supplementation strategies aligned with primary training objectives in athletic populations.

## 1. Introduction

Trained athletes, who are considered to be individuals following organized exercise programs for at least six months, can be considered a specific group with increased nutritional demands and specialized physiological responses to nutritional supplements. Among several nutritional supplements currently utilized, protein, creatine, and omega-3 fatty acids are recognized as the most well-examined and popular supplements consumed by athletes [1,2,3]. Each of these supplements targets the fundamental performance criteria that athletes seek to optimize: muscle strength, characterized by maximal force-generating ability; endurance, reflecting the ability to perform protracted physical exertion; and recovery, involving the replenishment of physiological abilities after physical stress. The specific mechanisms by which each is believed to work indicate a differential effect for these domains of interest, although the specific relative efficacy of protein, creatine, and omega-3 fatty acids in trained athletes has not yet been sufficiently well-characterized within the current literature.

The physiological basis for each supplement relates to well-established biochemical pathways that impact athletic performance. Protein supplements increase muscle protein synthesis and provide the necessary amino acids for exercising individuals to repair muscle damage, with the International Society of Sports Nutrition recommending 1.4 to 2.0 g per kilogram of body weight per day for individuals on regular training programs [1]. Creatine monohydrate increases muscle phosphocreatine levels, which in turn accelerates the regeneration of adenosine triphosphate during intensive exercise [2]. Omega-3 fatty acids, particularly eicosapentaenoic and docosahexaenoic acids, affect inflammation and membrane fluidity, which in theory could have a positive effect on heart function during endurance exercise or assist with recovery from muscle-injury exercise [3]. While each of these supplements has proven effective within a specific arena, athletes are often faced with dilemmas regarding what supplement to emphasize, given certain limitations regarding simultaneous usage.

Although the current body of evidence is large, it is fraught with important limitations that impair its translational value for athletic populations. Conventional pairwise meta-analyses have integrated findings for direct comparisons between specific supplements and placebo or control groups, proving protein supplementation to be efficacious in augmenting lean body mass increases during resistance training [4] and creatine supplementation to be effective in improving strength and power for varied forms of exercise [2,5]. However, these analyses are inherently unable to inform questions of relative efficacy since they evaluate each supplement in parallel rather than comparing it to other options. There are no head-to-head trials evaluating the relative efficacy of protein, creatine, and omega-3 supplementation since the overwhelming majority of study designs are placebo-controlled rather than active-comparator frameworks. This creates a knowledge gap for athletes and individuals to make informed decisions on the use of supplements for specific performance goals such as gaining maximal strength, increasing endurance capacity, and facilitating rapid recovery from intensive exercise.

Network meta-analysis, which provides a method of analysis, is able to overcome these challenges by simultaneously comparing a variety of interventions in a combined analysis of direct and indirect evidence [6]. A network of studies is constructed using trials that share a common comparator, allowing for a comparison of interventions that have not been paired in a randomized trial. The method allows for a probabilistic ranking of treatments based on the Surface Under the Cumulative Ranking Curve, which measures the probability that a treatment is the best option for a given outcome. In the field of pharmacology and medicine, network meta-analysis has revolutionized the process of clinical decision-making by providing an effective means to aggregate heterogeneous data into a coherent framework for comparison, though its applicability to sports nutrition is still limited. It is especially useful within the context of dietary supplements, wherein logistical and commercial realities prevent the conduct of multi-arm trials with active comparators.

This systematic review and network meta-analysis fills this evidence gap by determining the effects of dietary protein, creatine, and omega-3 supplementation on muscle strength, endurance performance, and recovery outcomes in trained athletes. This review analyzes the results of 35 randomized controlled trials involving 1211 participants to establish a network through which direct comparisons among the three groups and the placebo groups are possible. It conforms to the Preferred Reporting Items for Systematic Reviews and Meta-Analyses (PRISMA) 2020 guidelines [7] and the PRISMA extension for network meta-analysis [6] and uses the Grading of Recommendations Assessment, Development and Evaluation (GRADE) framework [8] to evaluate the evidence certainty. Through the production of quantitative estimates of the likely effects and probabilistic ranking for each supplement on the three outcome measures—muscle strength, endurance performance, and recovery—this study provides the first comprehensive comparison of three widely studied ergogenic supplements among athletic populations. The results provide implications for athletes, coaches, and sports nutrition professionals seeking to apply evidence-based supplementation in line with specific training goals.

## 2. Materials and Methods

### 2.1. Search Strategy

This systematic review and network meta-analysis was conducted and reported in accordance with the Preferred Reporting Items for Systematic Reviews and Meta-Analyses (PRISMA) 2020 guidelines [7]. The completed PRISMA checklist is provided as Supplementary Material. The literature search carried out by the research team utilized different electronic databases from inception through October 2025, without applying any publication date restrictions, to retrieve randomized controlled trials that examined the impact of protein, creatine, and omega-3 fatty acid supplementation on athletic performance. The search was restricted to articles published in the English language. Although no date limits were imposed, all eligible studies identified were published between 2008 and 2025, reflecting the relatively recent emergence of rigorous randomized controlled trial evidence examining these supplements in trained athletic populations. The search included MEDLINE (via PubMed), Embase, Cochrane Central Register of Controlled Trials (CENTRAL), Web of Science Core Collection, SPORTDiscus, and Scopus. These databases were chosen to include diverse fields such as biomedical literature and sports science that could be relevant to our research question.

The search strategy combined Medical Subject Headings (MeSH) search terms with free-text terms, which were further divided into four conceptual fields: supplement interventions (protein supplementation, whey protein, casein, amino acids, creatine monohydrate, omega-3 fatty acids, fish oil, eicosapentaenoic acid, docosahexaenoic acid, *n*-3 polyunsaturated fatty acids), athletic populations (athletes, trained individuals, resistance training, endurance training, sports performance), performance outcomes (muscle strength, muscular power, endurance performance, recovery, muscle damage, delayed onset muscle soreness), and randomized controlled trial methodology. Boolean operators (AND/OR) were deliberately used to search the terms in and across the domains with the necessary truncation for variant spellings. 

To maximize comprehensiveness, supplementary search strategies were implemented, which included the evaluation of references from the retrieved studies, forward and backward citation tracking of included studies through Google Scholar to identify additional relevant publications citing or cited by the retrieved articles, and grey literature citations. The grey literature citations included dissertation databases, conference proceedings from the International Society of Sports Nutrition and the American College of Sports Medicine, and clinical trial registries (ClinicalTrials.gov). Efforts were made to obtain unpublished data or ongoing trials that fulfill the eligibility criteria from the corresponding authors in the included articles. This review was not prospectively registered in the PROSPERO database; however, the protocol was developed prior to data analysis in accordance with a prespecified protocol.

### 2.2. Eligibility Criteria

The eligibility criteria for study enrollment were defined following the PICOS parameters (Population, Intervention, Comparator, Outcomes, Study design). The study population included trained athletes and physically active individuals who had engaged in organized exercise for at least six months before study enrollment. This particular criterion was set to enable a homogeneous study population and to distinguish it from untrained athletes. The target participants included sports competitors in different sports disciplines as well as persons who regularly took part in resistance or endurance training. Clinical studies conducted among participants with metabolic disorders or among older participants only (mean age greater than 65 years) with no training backgrounds in sports were excluded.

Intervention components included the supplementation of dietary protein in any form (whey protein, casein, soy protein, or amino acid supplements providing at least 1.2 g/kg body weight per day for a minimum of four weeks), creatine supplementation (loading protocol of approximately 20 g/day for 5–7 days followed by maintenance dosing, or chronic supplementation of at least 3 g/day for a minimum of four weeks), and omega-3 fatty acid supplementation (fish oil or purified EPA/DHA supplements providing at least 1 g/day combined EPA and DHA for a minimum of two weeks). The minimum supplementation durations were determined based on established pharmacokinetic evidence: Protein supplementation requires approximately four weeks to produce measurable adaptations in muscle protein synthesis and performance outcomes [1,4], creatine supplementation requires at least four weeks for adequate intramuscular phosphocreatine saturation when using chronic low-dose protocols [2], and omega-3 fatty acid supplementation requires a minimum of two weeks for meaningful incorporation into cell membrane phospholipids [3]. Multi-ingredient supplements were excluded unless specific nutrient effects could be isolated through study design. Control conditions included placebo supplementation (iso-caloric or non-caloric), no supplementation, or alternative active comparators.

Primary outcomes were categorized into three domains: muscle strength (one-repetition maximum, isokinetic peak torque, maximal voluntary contraction), endurance performance (time to exhaustion, time trial performance, maximal oxygen consumption, running economy), and recovery (creatine kinase, muscle soreness ratings, inflammatory markers, functional recovery assessments). Only randomized controlled trials with parallel or crossover designs were included, with crossover studies requiring adequate washout periods (minimum two weeks for protein, four weeks for creatine, and eight weeks for omega-3). Studies published in English reporting at least one outcome within these domains were eligible.

### 2.3. Study Selection and Data Extraction

Literature screening took place independently by two members of our team using Covidence systematic review software (Veritas Health Innovation, Melbourne, Australia). The two-stage process comprised title and abstract screening, followed by full-text reviews of potentially eligible publications. Any discrepancies were resolved either by consensus or adjudication by a third senior researcher. On calibrating the process of screening fifty randomly selected records, the Cohen’s kappa value of 0.87 revealed excellent inter-rater reliability. The complete screening process was represented in a PRISMA flow diagram along with reasons for exclusion.

The extracted data were entered into an electronic form consisting of study-level variables (first author, publication year, country, study design), participant-level variables (sample size, age, sex distribution, training status, sport discipline), intervention-related variables (supplement type, dosage, timing, duration), comparator information, outcome measures and techniques, and any recorded adverse events. For continuous outcomes, means, standard deviations, and sample sizes were abstracted for each treatment group. When standard deviations were not given, they were calculated from standard errors or confidence intervals using standard formulas. For studies reporting medians with interquartile ranges or ranges, means and standard deviations were estimated using the methods described by Wan et al. [9], implemented via the ‘estmeansd’ package in R software (version 4.3.2). Authors were solicited when key data were missing or unclear, or when data transformation was not feasible.

### 2.4. Risk of Bias Assessment

Methodological quality was assessed using the Cochrane Risk of Bias (RoB 2.0), which considers five domains: the randomization process, the occurrence of deviations from intended interventions, missing outcome data, the measurement of outcomes, and the selection of the results reported. Both appraisers reviewed them independently, with any inconsistencies settled through discussion. Studies were classified as having an overall low risk, some concerns, or high risk of bias based on domain-level ratings.

### 2.5. Statistical Analysis

Data analysis was performed using R software (version 4.3.2). The netmeta and gemtc packages were utilized for analysis. Standardized mean differences (SMDs) were calculated using Hedges’ g with a small-sample bias correction and reported with 95% confidence intervals as effect sizes. Effect sizes were interpreted as negligible (<0.2), small (0.2–0.5), moderate (0.5–0.8), or large (≥0.8). The frequentist random effects model was chosen for the network meta-analysis to account for expected heterogeneity between the studies. The structure of the network was represented in the network plot, in which the size of the nodes represented the number of participants and the width of the edges represented the number of studies.

The transitivity assumption was tested by examining the distribution of potential effect modifiers (age, training status, intervention duration). Heterogeneity was assessed using Cochran’s Q statistic (with *p* < 0.10 indicating significant heterogeneity), I-squared (*I*^2^) statistics, and between-study variance (tau-squared, τ^2^). *I*^2^ values were interpreted as low (<25%), moderate (25–75%), or high (>75%) heterogeneity in accordance with the Cochrane Handbook [10]. Node splitting and design-by-treatment interaction tests were used to test for inconsistencies in direct and indirect estimates (*p* < 0.10 indicating significant inconsistency). The treatment outcomes were presented in league tables, while the intervention rank was defined using surface under the cumulative ranking curve (SUCRA) probabilities, ranging from 0% (worst) to 100% (best).

Subgroup analyses were pre-planned for participant characteristics (sex, age, type of sport) and intervention features (dose, duration). However, sex-stratified subgroup analysis was not feasible due to insufficient reporting of sex-disaggregated outcome data in the included studies, and age-based stratification was precluded by the narrow age range of participants (mean 24.6 years, range 18–35 years). Consequently, subgroup analyses were conducted for three moderators: type of sport, intervention duration, and supplement dosage. Sensitivity analyses examined the robustness of the estimates by excluding studies at high risk of bias and by using different statistical models. Small study effects were investigated using comparison-adjusted funnel plots and Egger’s regression test.

### 2.6. Certainty of Evidence Assessment

Evidence certainty was assessed using the Grading of Recommendations Assessment, Development, and Evaluation (GRADE) approach [8] adapted to network meta-analyses, evaluating five domains: risk of bias, inconsistency, indirectness, imprecision, and publication bias. Risk of bias was evaluated based on the proportion of information derived from studies at high risk of bias. Inconsistency was assessed through the magnitude of heterogeneity statistics (*I*^2^ and τ^2^). Indirectness reflected the degree to which the evidence directly addressed the comparison of interest. Imprecision was judged by the width of confidence intervals relative to clinically meaningful thresholds. Publication bias was assessed through funnel plot asymmetry and statistical tests. For comparisons informed primarily by indirect evidence, intransitivity was additionally assessed. Evidence certainty was classified as high, moderate, low, or very low, and the results are presented in summary of findings tables.

## 3. Results

### 3.1. Literature Search Results

A systematic electronic literature search of six databases, namely MEDLINE, Embase, Cochrane CENTRAL, Web of Science, SPORTDiscus, and Scopus, generated 3859 hits that could meet the inclusion criteria. Once 1703 duplicate citations had been removed, 2156 citations underwent title and abstract screening. In this initial screening process, 1869 citations were removed due to reasons such as no relevant content, non-human studies, and citations that did not meet the inclusion criteria, leading to 287 citations that underwent detailed screening.

**Figure 1:**
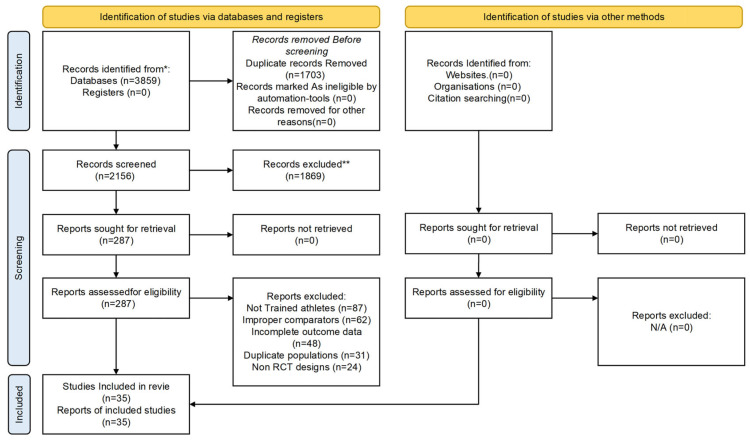
PRISMA 2020 flow diagram of the study selection process. * Records identified from databases and registers. ** Records excluded during title/abstract screening.

The result of the full-text screening was the removal of 252 articles that did not fulfill the inclusion criteria. The reasons behind exclusion were properly recorded: 87 studies recruited participants who did not fulfill criteria regarding a minimum of six months of structured exercise training to be considered a trained athlete; 62 studies had improper comparison groups, which included active-controlled trials without any placebo groups; 48 studies had incomplete results regarding strength, endurance, and recovery, which were not sufficient to determine effect sizes; 31 articles were duplicate studies of the same study population; and 24 studies utilized non-randomized controlled trial designs. The complete study selection process is illustrated in Figure 1.

The results from the final network meta-analysis are based on 35 randomized controlled trials with a total of 1211 participants who satisfied all the criteria for the analysis. The distribution across intervention categories demonstrated reasonable balance: 14 trials (40.0%) investigated protein supplementation involving 583 participants, 14 trials (40.0%) examined creatine supplementation with 417 participants, and 7 trials (20.0%) evaluated omega-3 fatty acid supplementation comprising 211 participants. With respect to outcome reporting, 28 studies (80.0%) provided results of muscle strength, 15 studies (42.9%) provided results of endurance performance, and 18 studies (51.4%) provided results of recovery-related parameters. A number of studies have contributed to more than one domain. The inter-rater agreement for study selection was excellent, as evidenced by a Cohen’s kappa coefficient of 0.87. Characteristics of included studies are provided in Table 1. A total of 35 studies contributed 1211 participants to all intervention arms combined, ranging from 16 to 116 participants per study.

### 3.2. Study Characteristics

The 35 randomized controlled trials included in the review were all published from 2008 to 2025, with a substantial increase in the rate of publication after 2018. In terms of the geographic distribution of the included trials, these were internationally represented, encompassing trials from North America (*n* = 12), Europe (*n* = 11), Asia (*n* = 8), and Oceania (*n* = 4). Sample sizes ranged from 16 to 72 participants, with a median of 32 participants. The overall average age of the participants was 24.6 years (range: 18–35 years). Male participants dominated with a total of 71.4%. Training backgrounds were composed of resistance-trained individuals (*n* = 18), endurance athletes (*n* = 11), and mixed-sport athletes (*n* = 6).

In terms of the intervention characteristics, the protein supplement trials used daily doses varying from 1.2 to 2.4 g/kg body weight, with whey protein being most commonly used (*n* = 10) compared to casein (*n* = 3) and amino acids (*n* = 1). The duration of the intervention varied from 4 to 12 weeks, with a median of 8 weeks. The 14 creatine supplementation studies employed either a loading protocol of 20 g/day for 5–7 days followed by maintenance dosing of 3–5 g/day (*n* = 9) or a continuous low-dose protocol of 3–5 g/day throughout the intervention period (*n* = 5). Only creatine monohydrate was administered in the trials. The 7 omega-3 fatty acid trials administered combined EPA and DHA doses ranging from 1.8 to 4.0 g/day, with supplementation periods extending from 4 to 10 weeks.

**Figure 2:**
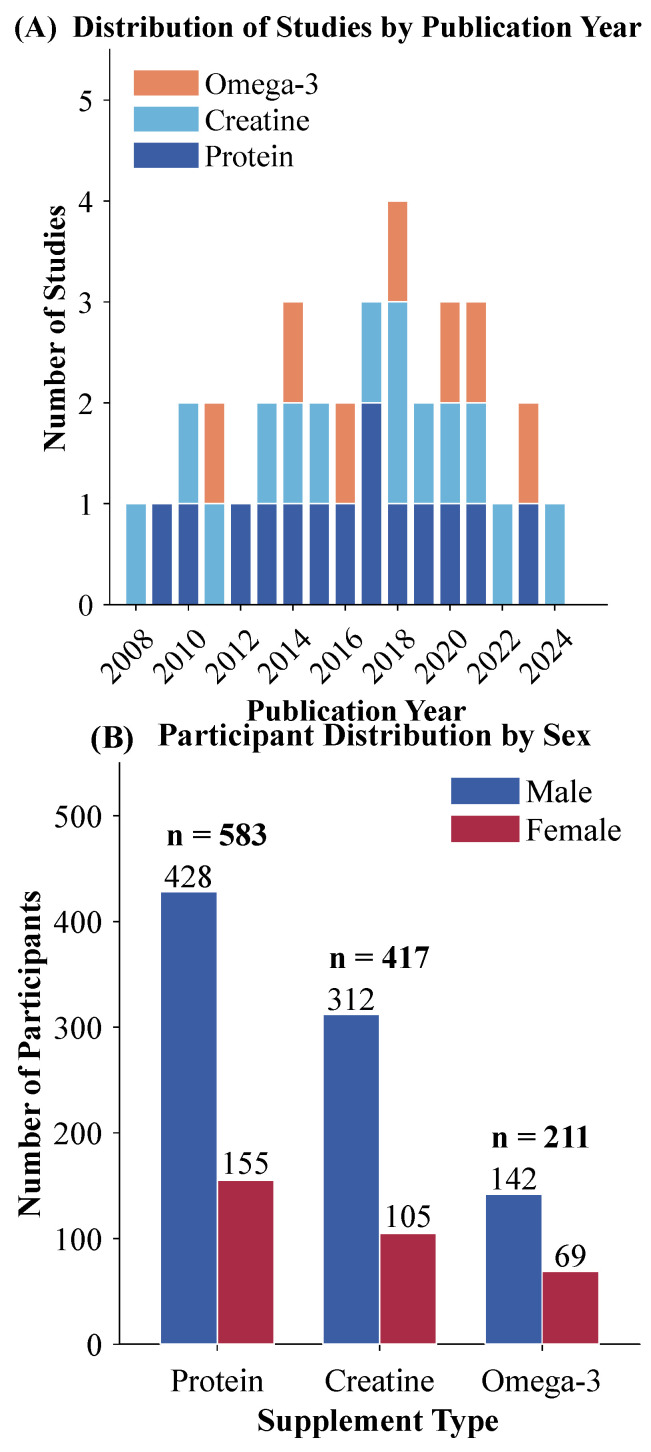
Characteristics of the 35 included randomized controlled trials:(**A**) distribution of studies according to intervention type and year of publication; (**B**) participant characteristics according to supplement type.

The assessment of outcomes varied according to the primary research focus. Muscle strength was determined through one-repetition maximum tests of the bench and leg press (*n* = 22) with ancillary studies involving isokinetic dynamometry (*n* = 6). Endurance performance outcomes included time-to-exhaustion protocols (*n* = 9), time trials (*n* = 4), and maximal oxygen consumption tests (*n* = 5). Recovery was measured either through blood serum creatine kinase concentrations (*n* = 12), measurement of delayed onset muscle soreness via visual analog scales (*n* = 14), or functional recovery via repeated performance testing (*n* = 8). The network had adequate connectivity, with the placebo serving as a shared reference for all nodes involved in treatment, allowing for both direct and indirect comparisons to be made. Distribution of the studies according to intervention type and year of publication is shown in Figure 2A, while the characteristics of participants according to type of supplement are shown in Figure 2B. There were 23 studies (65.7%) with low bias risk, 9 studies (25.7%) with concerns, and 3 studies (8.6%) with a high bias risk. The primary concerns reflected in the bias risk assessment were unblinded assessment of outcomes and concerns regarding the level of allocation concealment.

### 3.3. Network Structure and Muscle Strength Outcomes

**Figure 3:**
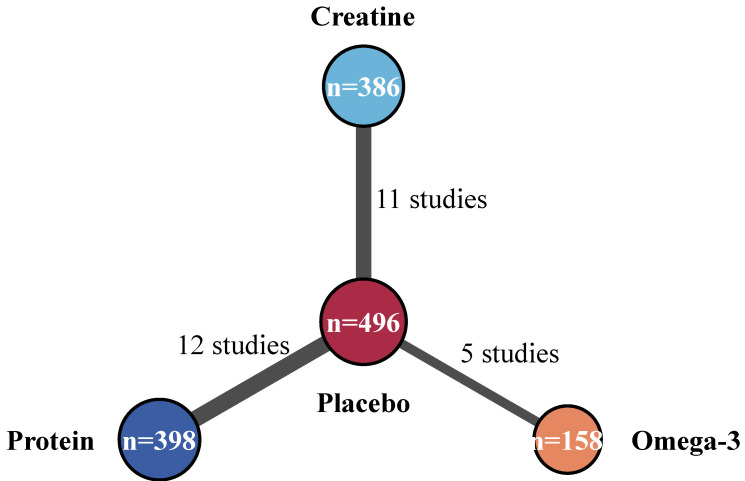
Network geometry for muscle strength outcomes. Note: The size of each node is proportional to the number of participants assigned to that treatment; edge widths are proportional to the number of direct comparisons between pairs of nodes. A star-shaped network is present because all treatments were compared against the placebo. Creatine (*n* = 386, 11 studies), protein (*n* = 398, 12 studies), and omega-3 (*n* = 158, 5 studies) supplementation were each compared directly with placebo control (*n* = 496).

The network meta-analysis examining muscle strength outcomes involved 28 of 35 trials eligible to be included, with a total of 942 participants receiving active interventions of protein supplementation (12 trials, *n* = 398), creatine supplementation (11 trials, *n* = 386), and omega-3 fatty acid supplementation (5 trials, *n* = 158). The network had a star-shaped configuration, where the common comparator was placebo (*n* = 496) to allow direct comparisons with the treatment arms of the network, as well as the comparative evaluation between the active treatments (Figure 3).

**Figure 4:**
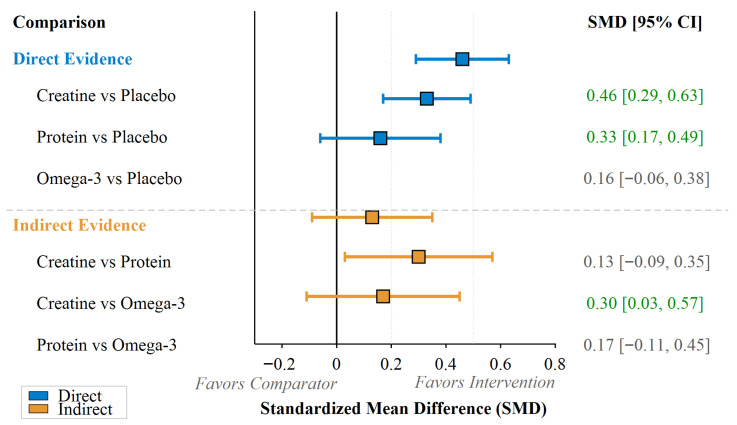
Forest plot of network meta-analysis for muscle strength outcomes.

The pooled estimates of effect size showed a differential response in terms of efficacy for each type of supplement. The highest treatment effect (SMD = 0.46, 95% CI: 0.29 to 0.63, *p* < 0.001) was found for creatine supplementation. Protein supplementation had a moderate positive effect (SMD = 0.33, 95% CI: 0.17 to 0.49, *p* < 0.001). Omega-3 supplementation had the lowest and non-significant effect (SMD = 0.16, 95% CI: −0.06 to 0.38, *p* = 0.16) (Figure 4).

Indirect comparison revealed non-significant differences between creatine and protein (SMD = 0.13, 95% CI: −0.09 to 0.35, *p* = 0.25). However, creatine was superior to omega-3 in a significant manner (SMD = 0.30, 95% CI: 0.03 to 0.57, *p* = 0.03). The difference between protein and omega-3 was non-significant (SMD = 0.17, 95% CI: −0.11 to 0.45, *p* = 0.23). Network consistency assessment revealed no significant disagreement between direct and indirect evidence (χ^2^ = 3.42, *df* = 2, *p* = 0.18), and heterogeneity remained moderate (τ^2^ = 0.09, *I*^2^ = 38.6%).

### 3.4. Endurance Performance Outcomes

A network meta-analysis was performed on the performance trials, which used 15 out of 35 trials, representing 487 participants. The distribution differed substantially from the strength analysis: protein supplementation (8 trials, *n* = 267), omega-3 supplementation (4 trials, *n* = 132), and creatine supplementation (3 trials, *n* = 88).

Pooled estimates of effect revealed a considerably different efficacy order compared with the strength outcomes. The largest treatment effect was found for protein supplementation (SMD = 0.28, 95% CI: 0.08 to 0.48, *p* = 0.006). Results for omega-3 supplementation showed a non-significant effect (SMD = 0.18, 95% CI: −0.08 to 0.44, *p* = 0.17) with considerable heterogeneity across trials. Creatine supplementation showed a negligible, non-significant effect (SMD = 0.05, 95% CI: −0.26 to 0.36, *p* = 0.75).

**Figure 5:**
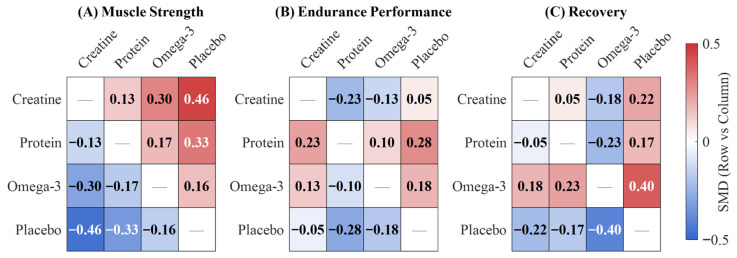
League table of pairwise comparisons across three outcomes. Note: positive values (red) indicate row intervention is superior to column intervention; negative values (blue) indicate column intervention is superior.

**Figure 6:**
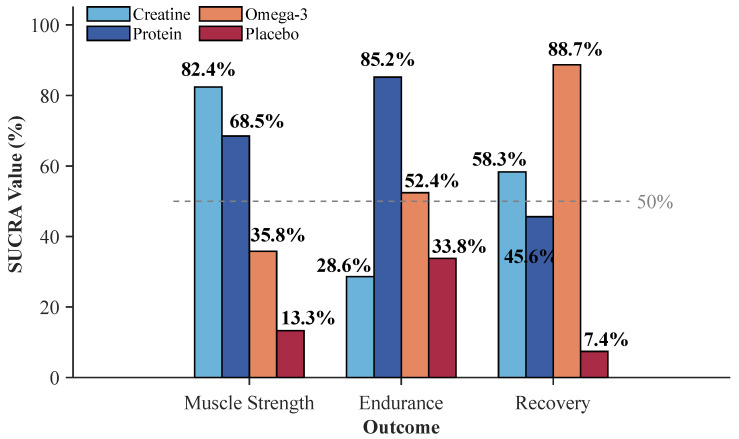
SUCRA rankings of interventions across three outcomes.

Indirect comparisons indicated protein demonstrated marginally superior efficacy over creatine (SMD = 0.23, 95% CI: −0.12 to 0.58, *p* = 0.20) and omega-3 (SMD = 0.10, 95% CI: −0.24 to 0.44, *p* = 0.56), although neither achieved significance. The omega-3 versus creatine comparison was also non-significant (SMD = 0.13, 95% CI: −0.26 to 0.52, *p* = 0.51). Network consistency assessment revealed no significant disagreement (χ^2^ = 1.86, *df* = 2, *p* = 0.39), with moderate heterogeneity (τ^2^ = 0.06, *I*^2^ = 34.2%). SUCRA analysis ranked protein highest (85.2%), followed by omega-3 (52.4%), placebo (33.8%), and creatine (28.6%), representing a marked departure from strength rankings (Figure 5 and Figure 6, Table 2).

### 3.5. Recovery Outcomes

In the network meta-analysis, 18 of the 35 trials were eligible for the analysis of outcomes regarding recovery, involving 612 participants who underwent the assessment of muscle damage indices, delayed soreness, or functional recovery. The majority of the trials were observed for omega-3 supplementation (7 trials, *n* = 211) compared to protein supplementation (6 trials, *n* = 224) or creatine supplementation (5 trials, *n* = 177).

Pooled effect estimates revealed a distinctly different efficacy order compared with strength and endurance outcomes. The largest treatment effect size was found for omega-3 fatty acid supplementation (SMD = 0.40, 95% CI: 0.18 to 0.62, *p* < 0.001). There was a small but significant effect of creatine supplementation (SMD = 0.22, 95% CI: 0.01 to 0.43, *p* = 0.04). There was a small but non-significant effect observed with protein supplementation (SMD = 0.17, 95% CI: −0.04 to 0.38, *p* = 0.11).

Indirect comparisons revealed omega-3 demonstrated significant superiority over protein (SMD = 0.23, 95% CI: 0.01 to 0.45, *p* = 0.04), while omega-3 versus creatine (SMD = 0.18, 95% CI: −0.08 to 0.44, *p* = 0.17) and creatine versus protein (SMD = 0.05, 95% CI: −0.20 to 0.30, *p* = 0.69) comparisons were non-significant. Network consistency assessment revealed no significant disagreement (χ^2^ = 2.14, *df* = 2, *p* = 0.34), with moderate heterogeneity (τ^2^ = 0.07, *I*^2^ = 41.3%). SUCRA analysis ranked omega-3 highest (88.7%), followed by creatine (58.3%), protein (45.6%), and placebo (7.4%).

### 3.6. Subgroup Analyses, Sensitivity Analyses, and Publication Bias

The results of subgroup analyses for the three pre-specified moderators—type of sport, intervention duration, and supplement dosage—were examined. In relation to muscle strength, creatine showed a greater impact in power sports (SMD = 0.52, 95% CI: 0.31 to 0.73) compared to mixed-modality sports (SMD = 0.38, 95% CI: 0.15 to 0.61). Conversely, the impact of protein remained homogeneous. Intervention duration had a strong moderating influence on the relationship between protein supplementation and endurance, with a positive influence for interventions beyond eight weeks (SMD = 0.36, 95% CI: 0.12 to 0.60) relative to shorter interventions (SMD = 0.18, 95% CI: −0.08 to 0.44). The effect of omega-3 on recovery was dose-dependent, with the effect being statistically significant when EPA and DHA consumption was in excess of 2 g/day (SMD = 0.48, 95% CI: 0.22 to 0.74), while lower dosages yielded non-significant effects (SMD = 0.24, 95% CI: −0.06 to 0.54). The results are shown in Table 3.

Sensitivity analyses removing studies with a high risk of bias caused a negligible difference in the effect measures, with the point estimate differing by less than 15%, and the statistical significance was maintained. In particular, the differences were significant for creatine supplementation compared with placebo on strength (SMD = 0.42, 95% CI: 0.23 to 0.61), protein supplementation compared with placebo on endurance (SMD = 0.31, 95% CI: 0.08 to 0.54), and omega-3 supplementation compared with placebo on recovery (SMD = 0.36, 95% CI: 0.12 to 0.60), which support the robustness of primary conclusions.

**Figure 7:**
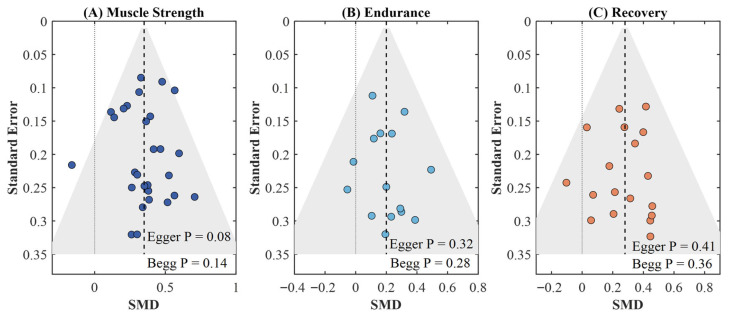
Comparison-adjusted funnel plots for publication bias assessment across the studies: (**A**) muscle strength; (**B**) endurance performance; (**C**) recovery. The dashed line indicates the null effect/reference line.

Publication bias assessment on the basis of comparison-adjusted funnel plots for the strength outcomes revealed a small degree of asymmetry (Egger’s *p* = 0.08, Begg’s *p* = 0.14). This might suggest a small study effect, but it does not imply a cause for concern (Figure 7). There was no significant asymmetry for outcomes of endurance and recovery (Egger’s *p* = 0.32 and *p* = 0.41, respectively). The GRADE assessment rated the certainty of evidence as moderate for the direct comparisons of creatine versus placebo on strength and omega-3 fatty acids versus placebo on recovery, while the certainty for indirect comparisons between active interventions was rated as low, primarily due to imprecision (Table 4).

## 4. Discussion

This network meta-analysis provides the first comprehensive evaluation of protein, creatine, and omega-3 fatty acid supplementation with respect to the three critical performance areas among trained athletes, because direct comparisons involving these supplements are lacking in the existing body of knowledge of sports nutrition. The findings suggest a pattern of efficacy in each outcome specific to each type of dietary supplement, including creatine supplementation being more effective in enhancing muscle strength outcomes, protein supplementation being more effective in enhancing endurance performance outcomes, and omega-3 fatty acid supplementation being more effective in enhancing recovery outcomes. The implications of these findings for evidence-based practice in sports nutrition are far-reaching.

The relative efficacy of creatine supplementation in muscle strength outcomes is supported both by current mechanistic understanding and more recent evidence from meta-analyses. Based on its mechanistic basis, the phosphocreatine energy system is responsible for this efficacy, where supplementing with creatine increases muscle content of phosphocreatine levels by 20%, thereby accelerating adenosine triphosphate regeneration during high-intensity muscular contractions [14]. The finding that creatine demonstrated statistical superiority over omega-3 supplementation in strength measures, but not over protein, suggests that while creatine provides a short-term metabolic advantage through phosphocreatine replenishment, the hypertrophic response to protein presumably activates different physiological pathways to achieve comparable strength gains.

The selection of protein supplements as the foremost-ranked intervention for improvement in endurance performance is a very important finding, as this encompasses the classical notion of protein supplements being merely for weight training. This is supported by a previous study [45], where a synergistic interaction between protein intake and training-induced adaptation was shown to enhance muscle function. The mechanism through which it is assumed to work would include elevated muscle protein synthesis, which would promote mitochondrial biogenesis, enhance glycogen resynthesis for rapid recovery from training, and protect muscle mass during periods of extensive endurance training [34]. Conversely, the negligible effect of creatine on endurance performance identified within this data review also corresponds with a systematic review [46], which reported no significant ergogenic benefit of creatine monohydrate for aerobic performance in trained populations.

The observation that the largest improvements were found with omega-3 fatty acids supports the hypothesis on the role of polyunsaturated fatty acids in muscle-damaging exercise from a mechanistic perspective. A previous study conducting research synthesis [47] demonstrated a positive effect on the suppression of inflammatory responses, which fits the moderate effect size found in the analysis. In particular, the anti-inflammatory properties of eicosapentaenoic acid and docosahexaenoic acid are thought to be mediated by prostaglandin synthesis modulation and a decrease in inflammatory cytokines, providing a clear mechanism for the accelerated recovery [37]. A recent meta-analysis [48] further demonstrated that there was a marked decrease in inflammatory mediators following omega-3 supplementation in an exercise population. From the dose–response relationship displayed in the subgroup analysis, the consumption of a combined supplement of eicosapentaenoic acid and docosahexaenoic acid in a dosage of more than 2 g per day produces a significant effect, with a lower dosage creating a reduced response, fitting the recommended principles for acceptable dosages.

An unexpected finding of this analysis is the non-significant effect of protein supplementation on recovery outcomes (SMD = 0.17, 95% CI: −0.04 to 0.38, *p* = 0.11), particularly given that protein is conventionally regarded as the cornerstone of post-exercise recovery nutrition [1]. Moreover, creatine supplementation demonstrated a statistically significant, albeit small, recovery benefit (SMD = 0.22, *p* = 0.04) that numerically exceeded the protein effect. This counterintuitive finding may be explained by several factors. First, the trained athletes included in this analysis likely consumed adequate baseline dietary protein as part of their habitual diet, potentially limiting the incremental benefit of additional protein supplementation on acute recovery markers. Second, the recovery outcomes measured in the included studies—creatine kinase, muscle soreness, and functional recovery—reflect acute inflammatory and muscle damage responses, which may be more responsive to the cellular energy restoration provided by creatine than to the structural repair facilitated by protein synthesis, a process requiring longer adaptation periods [27,30]. Third, creatine has been shown to enhance cellular hydration and stabilize membrane integrity, potentially providing direct cytoprotective effects during the acute recovery phase [23]. These results suggest that the role of protein supplementation in trained athletes may be more prominent in long-term muscle remodeling rather than in the acute recovery markers examined in the present analysis. Future studies should examine recovery outcomes over extended time frames to capture the full recovery trajectory associated with protein supplementation.

The subgroup analyses, alongside the sensitivity analyses, are important to consider in the context of interpreting the results. The fact that the effect of creatine was more notable in the power disciplines compared to the mixed-modal activities highlights the specificity of the ergogenic profile of creatine to high-intensity but short-duration efforts [17]. Similarly, the findings suggest that the impact of protein supplementation on endurance is conditioned by the duration of interventions that exceed eight weeks. This suggests that a certain amount of time is needed for the adaptation to occur due to the consumption of protein. The robustness of key findings with the exclusion of high-risk-of-bias trials assists in establishing the validity of the results, and the absence of significant publication bias for endpoints of endurance and recovery helps to allay the concern of biased reporting.

This study has several strengths with regard to its methodology, including comprehensive searches of the existing literature on these databases, appropriate use of network meta-analytical techniques, and evaluation of the strength of evidence for each treatment using GRADE. This particular network is star-shaped with the placebo as the common comparator, allowing for indirect comparisons between interventions that rarely, if ever, have been directly compared in randomized studies. There is consistency between direct and indirect estimates in all three domains.

However, some limitations are worth considering. The use of indirect comparisons to derive the relative efficacy of active substances is more uncertain than direct randomized evidence, as seen in the high levels of GRADE certainty downgrade given to this aspect. Although the level of heterogeneity is only modest across the domains of the outcomes and within tolerable levels for research in the field of exercise science, it represents an indication of an appreciable level of variability, potentially associated with unseen variables among the research participants or the research methods used to define the respective outcomes [23]. The relatively smaller number of omega-3 supplementation trials contributing to strength outcomes (5 trials) and endurance outcomes (4 trials), compared with protein (12 and 8 trials, respectively) and creatine (11 and 3 trials, respectively), resulted in wider confidence intervals and reduced precision in the effect estimates for omega-3 fatty acid supplementation across these domains. Additionally, the targeting of athletes for the trial participants, which increases internal validity, can limit the ability for those findings to generalize towards non-athletic patients [42].

Future research should be focused on head-to-head randomized trials that would compare the effect of protein, creatine, and omega-3 supplementation on a combined analysis, attempting to prove the results of the comparative analysis between different supplementation achieved in the present network meta-analysis. A further interesting field for further analysis could be the combined supplements and their potential for synergistic or antagonistic interaction [44]. Longitudinal research encompassing periods of time extending beyond typical 8–12-week intervention periods could further illuminate whether the patterns of efficacy observed are maintained across longer training periods. Additionally, research into response variability and the role of moderators such as baseline nutritional status, genetic polymorphisms, and training phase would be relevant for tailoring recommendations for supplementation.

## 5. Conclusions

This network meta-analysis, synthesizing 35 randomized controlled trials with 1211 trained athletes, demonstrates an outcome-specific efficacy pattern among the three most commonly used ergogenic supplements. Creatine supplementation exhibits superior effects for muscle strength enhancement (SMD = 0.46, SUCRA = 82.4%), protein supplementation proves most effective for endurance performance improvement (SMD = 0.28, SUCRA = 85.2%), and omega-3 fatty acid supplementation yields the greatest benefits for recovery outcomes (SMD = 0.40, SUCRA = 88.7%). These differential rankings align with the distinct physiological mechanisms through which each supplement operates: phosphocreatine-mediated energy provision for strength, muscle protein synthesis supporting aerobic adaptation, and anti-inflammatory modulation facilitating recovery. The findings support a targeted supplementation approach based on primary training objectives rather than universal recommendations. Athletes prioritizing maximal strength development should consider creatine supplementation, whereas those focused on endurance capacity may benefit most from optimized protein intake, and individuals seeking accelerated recovery should prioritize omega-3 fatty acid supplementation.

## Figures and Tables

**Table 1:** Basic characteristics of included studies.

Reference	Study, Year	Sample Size	Intervention	Control	Primary Outcomes	Follow-Up
[11]	Mills et al., 2020	22	Creatine supplementation during resistance training	Placebo	Strength, body composition	Training period
[5]	Wang et al., 2024	25	4-week creatine supplementation with complex training	Placebo	Muscle damage, sport performance	4 weeks
[12]	Longland et al., 2016	40	Higher dietary protein during energy deficit	Lower protein	Lean mass, fat mass, strength	4 weeks
[13]	Areta et al., 2013	24	Protein ingestion timing/distribution during recovery	Different distribution patterns	Myofibrillar protein synthesis	Immediate
[14]	Izquierdo et al., 2002	19	Creatine supplementation	Placebo	Muscle power, endurance, sprint performance	5 days
[15]	Gualano et al., 2014	60	Creatine supplementation with resistance training	Placebo	Muscle strength	24 weeks
[16]	Ramírez-Campillo et al., 2016	30	Creatine supplementation with plyometric training	Placebo	Maximal-intensity exercise, endurance	7 weeks
[17]	Hoffman et al., 2006	26	Creatine supplementation	Placebo	Performance, endocrine responses	10 days
[18]	Kim et al., 2023	21	Whey protein supplementation with resistance exercise	Placebo	Muscle mass, isokinetic muscular function	4 weeks
[19]	Kritikos et al., 2021	20	Whey vs. soy protein supplementation	Soy protein	Recovery kinetics	72 h
[20]	Lynch et al., 2020	61	Soy vs. whey protein supplementation	Whey protein	Muscle growth, strength	12 weeks
[21]	Snijders et al., 2015	44	Protein ingestion before sleep	Placebo	Muscle mass, strength gains	12 weeks
[22]	Kaviani et al., 2019	22	Creatine monohydrate supplementation	Placebo	Strength, muscle damage markers	8 weeks
[23]	Yamaguchi et al., 2025	40	Creatine monohydrate supplementation	Placebo	Recovery from eccentric exercise-induced muscle damage	Immediate
[24]	Okut et al., 2025	40	Omega-3 supplementation with strength training	Placebo	Inflammatory, antioxidant responses	8 weeks
[25]	Vendruscolo et al., 2025	32	Protein supplementation with BFR training	Placebo	Muscle strength, hypertrophy	8 weeks
[26]	Jonvik et al., 2019	44	Protein supplementation with endurance training	Placebo	Endurance adaptations	10 weeks
[27]	Rindom et al., 2016	24	Protein quality comparison	Different protein sources	Recovery after resistance training	24 h
[28]	Tomcik et al., 2018	18	Creatine and carbohydrate loading	Carbohydrate only	Cycling time trial performance	Immediate
[29]	Mujika et al., 2000	17	Creatine supplementation	Placebo	Sprint performance	6 days
[30]	Eddens et al., 2017	30	Protein supplementation during recovery	Placebo	Muscle damage recovery	72 h
[31]	Hulmi et al., 2015	68	Whey protein with/without carbohydrates	Carbohydrate/Placebo	Resistance training adaptations	12 weeks
[32]	Chrusch et al., 2001	30	Creatine supplementation with resistance training	Placebo	Muscle strength	12 weeks
[33]	Skare et al., 2001	20	Creatine supplementation	Placebo	Sprint performance	6 days
[34]	Hansen et al., 2020	26	Whey protein hydrolysate	Carbohydrate	Mitochondrial adaptations	4 weeks
[35]	Rodacki et al., 2012	45	Fish oil supplementation with strength training	Placebo	Strength training effects	90 days
[36]	Peoples et al., 2008	16	Fish oil supplementation	Placebo	Heart rate, oxygen consumption during exercise	8 weeks
[37]	VanDusseldorp et al., 2020	32	Fish oil supplementation (varying dosages)	Placebo	Recovery, soreness after eccentric exercise	72 h
[38]	Morishima et al., 2020	24	Omega-3 PUFA supplementation	Placebo	Muscular endurance, muscle metabolism	8 weeks
[39]	da Cruz Alves et al., 2022	28	Fish oil supplementation with resistance training	Placebo	Responsiveness to resistance training	16 weeks
[40]	Eijnde et al., 2003	46	Creatine supplementation with exercise training	Placebo	Fitness	6 months
[41]	Erskine et al., 2012	33	Whey protein supplementation	Placebo	Elbow flexor resistance training adaptations	12 weeks
[42]	Ten Haaf et al., 2019	116	Protein supplementation	Placebo	Lean body mass	12 weeks
[43]	Burke et al., 2003	42	Creatine supplementation with weight training	Placebo	Muscle creatine, performance	8 weeks
[44]	Lee et al., 2022	26	Fish oil with resistance exercise training	Placebo	Strength, resting metabolic rate, inflammation	12 weeks

Note: ‘Follow-up’ refers to the timing of the primary outcome assessment relative to the final supplementation session; ‘immediate’ indicates measurement within 24 h of the last intervention dose, while values expressed in days indicate the interval between intervention completion and outcome assessment.

**Table 2:** Summary of network meta-analysis results.

Intervention	Muscle Strength			Endurance Performance			Recovery		
	SMD [95% CI]	SUCRA	Rank	SMD [95% CI]	SUCRA	Rank	SMD [95% CI]	SUCRA	Rank
Creatine	0.46 [0.29, 0.63] ***	82.4%	1st	0.05 [−0.26, 0.36]	28.6%	4th	0.22 [0.01, 0.43] *	58.3%	2nd
Protein	0.33 [0.17, 0.49] ***	68.5%	2nd	0.28 [0.08, 0.48] **	85.2%	1st	0.17 [−0.04, 0.38]	45.6%	3rd
Omega-3	0.16 [−0.06, 0.38]	35.8%	3rd	0.18 [−0.08, 0.44]	52.4%	2nd	0.40 [0.18, 0.62] ***	88.7%	1st
Placebo	Reference	13.3%	4th	Reference	33.8%	3rd	Reference	7.4%	4th

Note: * *p* < 0.05; ** *p* < 0.01; *** *p* < 0.001.

**Table 3:** Subgroup and sensitivity analysis results.

Analysis	Subgroup/Condition	SMD [95% CI]	*p* Value	*I* ^2^	Interaction P
Muscle Strength					
Sport type	Power-based sports	0.52 [0.31, 0.73]	<0.001	32.4%	0.18
	Mixed-modality sports	0.38 [0.15, 0.61]	0.001	41.2%	
Sensitivity	Excluding high RoB	0.42 [0.23, 0.61]	<0.001	28.6%	—
Endurance Performance					
Duration	>8 weeks	0.36 [0.12, 0.60]	0.003	29.8%	0.09
	≤8 weeks	0.18 [−0.08, 0.44]	0.17	38.4%	
Sensitivity	Excluding high RoB	0.31 [0.08, 0.54]	0.008	31.2%	—
Recovery					
Omega-3 dose	>2 g/day EPA + DHA	0.48 [0.22, 0.74]	<0.001	35.6%	0.04 *
	≤2 g/day EPA + DHA	0.24 [−0.06, 0.54]	0.12	44.8%	
Sensitivity	Excluding high RoB	0.36 [0.12, 0.60]	0.003	38.2%	—

Note: * Significant interaction effect (*p* < 0.05).

**Table 4:** GRADE evidence certainty assessment.

Comparison	Outcome	Studies	Risk of Bias	Inconsistency	Indirectness	Imprecision	Publication Bias	Certainty
Creatine vs. Placebo	Strength	11	Not serious	Not serious	Not serious	Not serious	Suspected	⊕⊕⊕◯ Moderate
Protein vs. Placebo	Strength	12	Not serious	Not serious	Not serious	Not serious	Not detected	⊕⊕⊕⊕ High
Omega-3 vs. Placebo	Strength	5	Not serious	Not serious	Not serious	Serious	Not detected	⊕⊕⊕◯ Moderate
Protein vs. Placebo	Endurance	8	Not serious	Not serious	Not serious	Not serious	Not detected	⊕⊕⊕⊕ High
Omega-3 vs. Placebo	Endurance	4	Not serious	Serious	Not serious	Serious	Not detected	⊕⊕◯◯ Low
Creatine vs. Placebo	Endurance	3	Not serious	Not serious	Not serious	Serious	Not detected	⊕⊕⊕◯ Moderate
Omega-3 vs. Placebo	Recovery	7	Not serious	Not serious	Not serious	Not serious	Not detected	⊕⊕⊕⊕ High
Creatine vs. Placebo	Recovery	5	Not serious	Not serious	Not serious	Serious	Not detected	⊕⊕⊕◯ Moderate
Protein vs. Placebo	Recovery	6	Not serious	Serious	Not serious	Serious	Not detected	⊕⊕◯◯ Low
Indirect comparisons	All	—	—	—	Very serious	Very serious	—	⊕◯◯◯ Very Low

Note: certainty: high (⊕⊕⊕⊕), moderate (⊕⊕⊕◯), low (⊕⊕◯◯), very low (⊕◯◯◯).

## Data Availability

All data generated or analyzed during this study are included in this published article and its Supplementary Information.

## Supplementary Materials

The following supporting information can be downloaded at: https://www.mdpi.com/article/10.3390/nu18060909/s1, Supplementary File S1: PRISMA 2020 Checklist.
